# Mucinous, endometrioid, and serous ovarian cancers with peritoneal dissemination are potent candidates for P-cadherin targeted therapy: a retrospective cohort study

**DOI:** 10.1186/s12885-020-07737-w

**Published:** 2021-01-07

**Authors:** Kayo Kayahashi, Yasunari Mizumoto, Ayumi Matsuoka, Takeshi Obata, Junpei Iwadare, Mitsuhiro Nakamura, Takiko Daikoku, Hiroshi Fujiwara

**Affiliations:** 1 Department of Obstetrics and Gynecology, Graduate School of Medical Sciences, Kanazawa University, 13-1, Takaramachi, Kanazawa, Ishikawa 920-8641 Japan; 2 Institute for Experimantal Animals, Advanced Science Research Center, Kanazawa University, 13-1, Takaramachi, Kanazawa, Ishikawa 920-8641 Japan

**Keywords:** P-cadherin expression, Ovarian cancer, Antibody-drug conjugate, Target therapy

## Abstract

**Background:**

Aberrant expression of P-cadherin has been reported in various cancers, and has been attracting attention as a target for cancer treatment. Ovarian cancer, the leading cause of death among gynecologic malignancies, is classified into four histological subtypes: serous, mucinous, endometrioid, and clear cell, and each has distinct biological behavior. Although a negative survival impact in serous ovarian cancer patients and some functional role in peritoneal dissemination have been reported, differences of P-cadherin expression in histological subtypes and the proportion and distribution of positive cells remain to be investigated. The aims of this study were to clarify the histological and distributional profiles of P-cadherin expression in ovarian cancer for development of target-therapy in near future.

**Methods:**

A total of 162 primary, 60 metastatic, and 8 recurrent tumors (all cases from 162 ovarian cancer patients) were enrolled in the study. Immunohistochemistry was performed for P-cadherin expression. Associations with clinicopathological characteristics and survival were analyzed.

**Results:**

P-cadherin expression showed a strong correlation with the FIGO stage, histological subtypes, positive peritoneal dissemination (*P* < 0.01), positive distant metastasis (*P* < 0.05), and trend toward negative overall survival probability (*P* = 0.050). P-cadherin was intensely and broadly expressed in mucinous, endometrioid, and serous subtypes (*P* < 0.01). Disseminated tumors demonstrated similar P-cadherin expression to primary tumors whereas metastatic lymph nodes demonstrated significantly decreased expression (*P* < 0.01).

**Conclusions:**

Mucinous, endometrioid, and serous ovarian cancer patients accompanied with peritoneal disseminations are the most potent candidates for P-cadherin targeted drug delivery strategies. P-cadherin-targeted therapy may benefit and improve survival of poor-prognosis populations.

**Supplementary Information:**

The online version contains supplementary material available at 10.1186/s12885-020-07737-w.

## Background

Epithelial ovarian cancer is associated with the highest mortality among gynecologic cancers [1]. Approximately 60–70% of patients are detected in an advanced stage (stages III and IV) at the time of diagnosis, because ovarian cancer rarely disseminates through the vasculature but has a tendency to metastasize to the peritoneum, unlike many other types of solid tumors [2] [3]. These patients with peritoneal dissemination at diagnosis have a 5-year survival rate of less than 30% and a very high recurrence rate even with a successful response to surgery or chemotherapy [1] [2] [4]. In recent years, successful improvement of survival with newly developed therapeutic agents, such as anti-angiogenic agents and poly ADP ribose polymerase (PARP) inhibitors, has been reported [5] [6] [7]. However, the mortality rate of ovarian cancer patients is still far from satisfactory, and so a new therapeutic strategy is warranted.

P-cadherin, a member of classical cadherin superfamily, is a cell adhesion factor first reported in 1986 as a new subtype of the cadherin found in mouse embryonic development [8]. Besides its regulatory role in implantation, embryo morphogenesis, cellular homeostasis, cell differentiation, cell shape, cell polarity, and growth and migration in fetal development [8] [9] [10], aberrant expression in various cancers has been reported. Since it is only faintly expressed in limited organs of normal adult tissue, it has been attracting attention as a therapeutic target.

Some studies on P-cadherin and ovarian cancer have been reported. Decreased E-cadherin and increased P-cadherin expression, so-called cadherin switching, has been observed when a tumor progresses from stage I to II, indicating its functional role at the stage of cancer spreading from the primary lesion to pelvic cavity [11] [12]. High expression of P-cadherin was reported to have a negative impact on survival in patients with high-grade serous subtypes of ovarian cancer [12]. Moreover, using the mouse ovarian cancer xenograft model, inhibition of P-cadherin by RNAi resulted in decreased peritoneal implantation [3]. These reports suggest the involvement of P-cadherin in ovarian cancer progression and the effectiveness of developing treatment targeting P-cadherin for ovarian cancer; however, the precise profile of P-cadherin expression in terms of histological subtypes, and the proportion and distribution of positive cancer cells in metastatic lesions and association with clinicopathological characteristics, are largely unknown. The aims of this study were to provide the expression profiles of P-cadherin in ovarian cancer, which are mandatory for development of targeted therapy in near future.

## Methods

### Patients and tissue samples

We recruited 162 patients with epithelial ovarian cancer who underwent primary debulking surgery or exploratory laparotomy between 2008 and 2018 at Kanazawa University Hospital, Japan. Patients from whom tissue from the primary lesion at the time of initial surgery was not obtained were excluded from this study. Of the 162 patients with ovarian cancer, 72 were classified in stage I, 16 in stage II, 64 in stage III, and 10 in stage IV. The median follow-up period after surgery was 47.0 months (range: 0.5–141.7). Formalin-fixed paraffin-embedded blocks of 162 primary lesions, 60 metastatic lesions, and eight recurrent tumors of 162 ovarian cancer patients were retrieved from the Department of Pathology, Kanazawa University Hospital, Japan. The study protocol was approved by the ethical committee of Kanazawa University and preoperative informed consent was obtained from the patients.

### Immunohistochemical staining and scoring

**Fig. 1:**
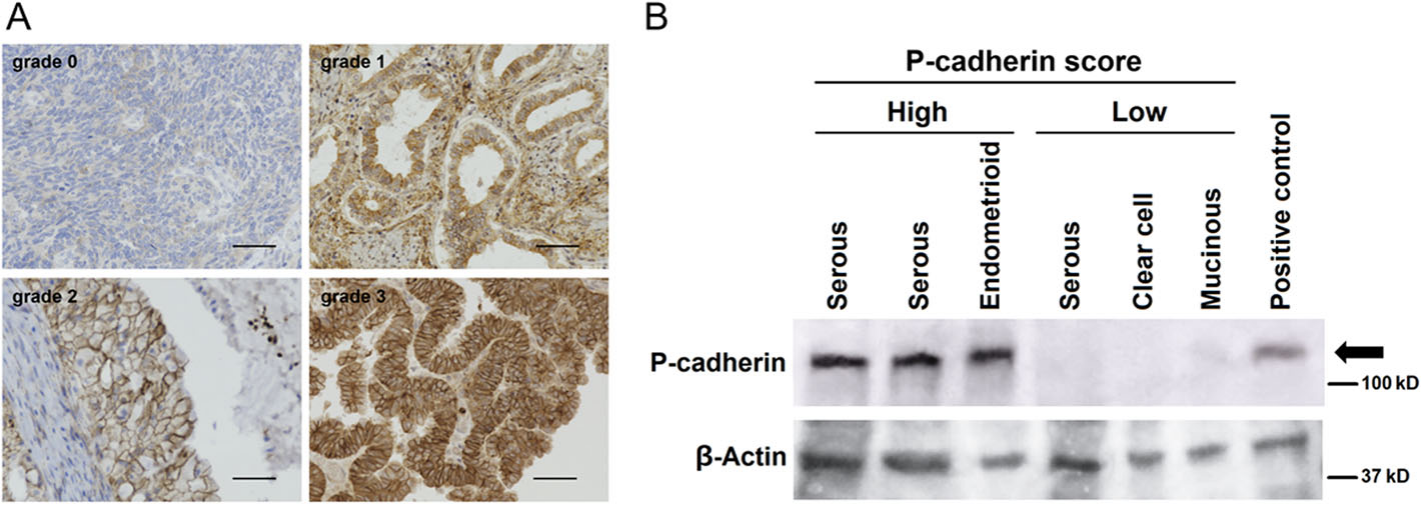
**a**: P-cadherin Intensity Score (IS); P-cadherin expression was scored from 0 to 3 based on the staining intensity. P-cadherin expression appears mainly in the membrane of tumor cells. Bars show 50 μm. **b**: Validation of P-cadherin antibody; Extracts from P-cadherin High and Low tumors were immunoblotted. Extract of chorionic villi was used as a positive control. The images of original blots were cropped and edited by PhotoDirector10 (CyberLink, Tokyo, Japan). The original full-length blots are presented in Supplementary Figures 2 and 3

Immunohistochemical staining (IHC) was performed on 3-μm, formalin-fixed, paraffin-embedded sections. Each section was deparaffinized in xylene and rehydrated in ethanol, and antigen retrieval was subsequently performed for 15 min. The slides were immersed in 0.3% hydrogen peroxide for 20 min to block endogenous peroxidase activity and then washed in 0.05 M phosphate-buffered saline (PBS, pH 7.4). After blocking, the slides were incubated with mouse monoclonal antibodies against P-cadherin (clone 56; BD Bioscience) at a concentration of 2.5 μg/mL (a dilution of 1:100) overnight at 4 °C. After washing, the sections were incubated for 30 min with biotin-labeled horse anti-mouse IgG at room temperature. Consequently, sections were treated with the avidin-biotin complex (VECSTATIN ABC kit; Vector Laboratories, Burlingame, USA) at room temperature. Sites of peroxidase activity were visualized with diaminobenzidine (Liquid DAB+ Substrate Chromogen System; Dako, Carpinteria, USA). After being counterstained with hematoxylin, sections were dehydrated and mounted. Expression analysis of proteins in malignant cells was performed by comparing staining intensity and the percentage of immunoreactive cells. Staining intensity was scored (IS: intensity score) on a scale of four grades: 0 (no staining of cancer cells), 1 (weak staining), 2 (moderate staining), and 3 (strong staining) (Fig. 1a), and the positive percentage score (PPS) was determined by the rate of stained cancer cells as follows: 0 (0%), 1 (1 to 10%), 2 (11 to 50%), and 3 (> 50%). The sum of IS and PPS was termed the “P-cadherin score”, and a P-cadherin score of 4 or higher was defined as a “high” score whereas 3 or lower was defined as “low”. The accuracy of antibody testing was confirmed by Western blotting. An extract of chorionic villi was used as a positive control, as previously reported [13].

### Statistical analysis

All statistical analyses were performed with EZR (Saitama Medical Center, Jichi Medical University, Saitama, Japan), which is a graphical user interface for R (The R Foundation for Statistical Computing, Vienna, Austria) [14]. More precisely, it is a modified version of R commander designed to add statistical functions frequently used in biostatistics. The association between the immunoreactive markers and clinicopathological features was analyzed using the χ^2^ test. The survival rates were assessed by the Kaplan-Meier method and compared by the log-rank test. To estimate risk factors for recurrence and survival by Cox proportional hazards regression analysis, continuous variables were converted into binary values. Continuous variables were compared using the Kruskal-Wallis test and the Wilcoxon signed-rank test, and are presented as the median and interquartile range. *P* < 0.05 was considered significant.

## Results

### P-cadherin expression is strongly correlated with histologic subtype and progressive clinicopathological features of ovarian cancer

**Fig. 2:**
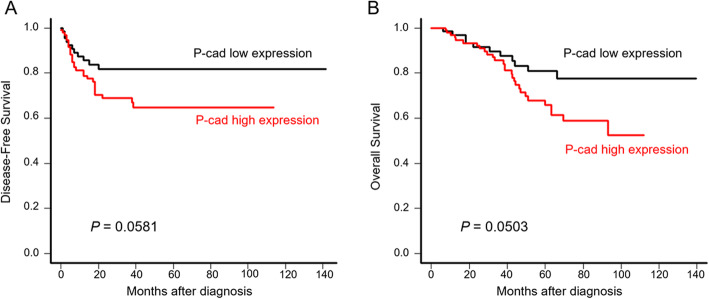
Cumulative disease-free survival and overall survival of ovarian cancer patients according to P-cadherin expression. Patients with a P-cadherin score of 0 to 3 were classified as showing Low expression, and patients with a score of 4 or more were classified as showing High expression. Kaplan-Meier curves showed a trend toward unfavorable survival in the High expression group in DFS (**a**, *P* = 0.0593) and OS (**b**, *P* = 0.0503)

We investigated the association between P-cadherin expression and clinicopathological features of ovarian cancer using IHC in 162 patients with ovarian cancer. IHC was performed on 162 primary lesions, 60 metastatic lesions, and 8 recurrent lesions. In the primary lesions, high expression of P-cadherin was observed in 95 of 162 cases (58.6%) (Table 1). P-cadherin was expressed on the cell membrane and in the cytoplasm, but not nucleus, of tumor cells (Fig. 1a). P-cadherin expression was significantly correlated with the International Federation of Gynecology and Ostetrics (FIGO) stage (*P* = .0024), peritoneal dissemination (*P* < .001), distant-site metastasis (*P =* .048), and histologic type (*P* < .001) (Table 1). As assessed by Kaplan-Meier survival analysis, patients with high expression of P-cadherin, although not significant, showed a trend toward a negative survival probability, as indicated by disease-free survival (DFS) and overall survival (OS) (*P* = .058, Fig. 2a; *P* = .050, Fig. 2b). Univariate analysis revealed that CA125, stage, peritoneal dissemination, and histologic type were indicators of DFS and OS. In multivariate analysis, CA125, stage, peritoneal dissemination, and histologic type, but not P-cadherin expression, were found to be independent prognostic factors (Table 3).

**Table 1:** Immunohistochemistry score of P-cadherin in each histological subtype

A: Intensity score (IS)					
Histologic type		Intensity score (%)			
	n	0	1	2	3
Clear cell	34	18 (52.9)	13 (38.2)	3 (8.8)	0 (0)
Endometrioid	30	1 (3.3)	8 (26.7)	16 (53.3)	5 (16.7)
Mucinous	23	5 (21.7)	7 (30.4)	10 (43.5)	1 (4.4)
Serous	75	7 (9.3)	14 (18.7)	44 (58.7)	10 (13.3)
All patients	162	31 (19.1)	42 (25.9)	73 (45.1)	16 (9.9)
B: Positive percent score (PPS)					
Histologic type		Positive percentage score (%)			
	n	0	1	2	3
Clear cell	34	18 (52.9)	6 (17.6)	6 (17.6)	4 (11.8)
Endometrioid	30	1 (3.3)	7 (23.3)	2 (6.7)	20 (66.7)
Mucinous	23	5 (21.7)	3 (13.0)	4 (17.4)	11 (47.8)
Serous	75	7 (9.3)	6 (8.0)	9 (12.0)	53 (70.7)
All patients	162	31 (19.1)	22 (13.5)	21 (13.0)	88 (54.3)
C: P-cadherin score (Sum of IS and PPS)					
Histologic type			Total score (%)		
		n	Low	High	
Clear cell		34	30 (88.2)	4 (11.8)	
Endometrioid		30	9 (30.0)	21 (70.0)	
Mucinous		23	12 (52.2)	11 (47.8)	
Serous		75	16 (21.3)	59 (78.7)	
All patients		162	67 (41.4)	95 (58.6)	

Immunohistochemistry score of P-cadherin in each histological subtype. **a**: intensity score, **b**: positive percentage score, **c**: P-cadherin score

### P-cadherin was intensely and broadly expressed in endometrioid and serous subtypes of ovarian cancer

**Fig. 3:**
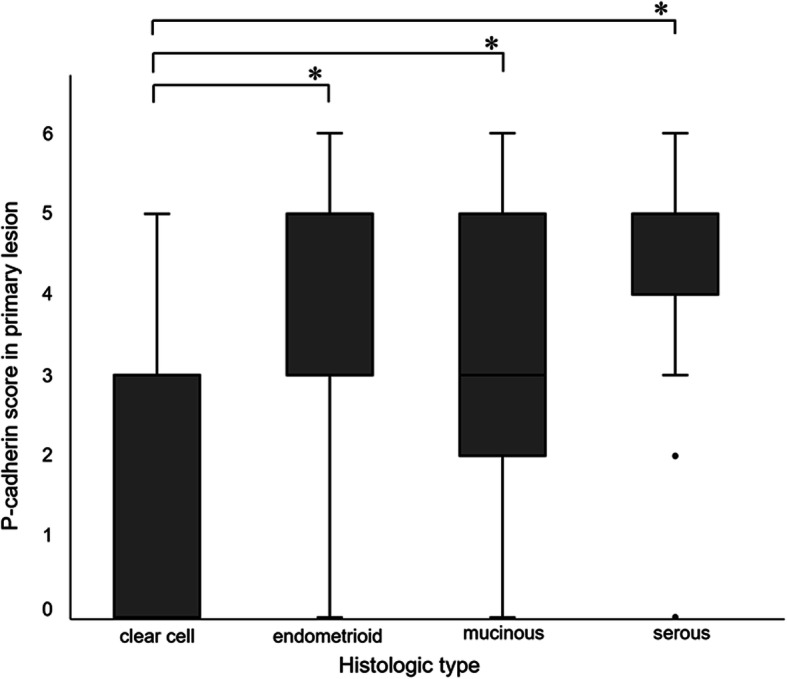
Differences in P-cadherin scores were compared among histologic subtypes. The P-cadherin score was assessed by the sum of the Intensity Score (IS) and Positive Percentage Score (PPS) in primary tumors of 162 patients. *P < 0 .01

We examined the difference in P-cadherin expression due to differences in histologic subtype. Tissue samples from primary lesions were obtained in 34 cases of clear cell carcinoma, 30 cases of endometrioid carcinoma, 23 cases of mucinous carcinoma, and 75 cases of serous carcinoma. P-cadherin scores for each histological subtype were compared by the Kruskal-Wallis test. A significant difference in the P-cadherin score was confirmed due to the difference in histological subtypes. Especially, endometrioid, serous, and mucinous carcinoma showed significantly high P-cadherin scores whereas the clear cell subtype showed significantly low P-cadherin scores (Fig. 3). Regarding the intensity score (IS), 21/30 (70%) and 54/70 (72%) of cases expressed IS 2 or more in endometrioid and serous subtypes respectively, whereas mucinous carcinoma demonstrated IS 1 or lower in 11/23 (47.9%) (Table 1a). On the other hand, regarding the positive percent score (PPS), 20/30 (66.7%) and 53/73 (70.7%) cases of endometrioid and serous subtypes, demonstrated P-cadherin expression in over 50% of cancer cells, respectively (Table 1b). These results indicate intense and widespread expression of P-cadherin in serous and endometrioid subtypes of ovarian cancer.

### Expression level of P-cadherin in primary tumor was preserved in peritoneal dissemination but reduced in metastatic lymph nodes

**Fig. 4:**
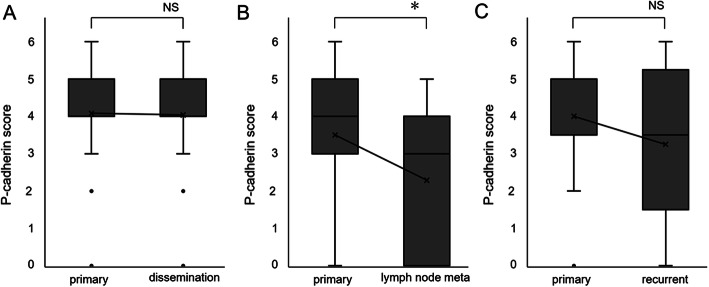
P-cadherin scores in primary, metastatic, and recurrent lesions were analyzed. **a**: P-cadherin score of primary tumor and peritoneal dissemination were compared (*n* = 49, *P* = .89). **b**: P-cadherin score of primary tumor and metastatic lymph nodes were compared (*n* = 14, *P = .0079). **c**: P-cadherin score of primary and recurrent tumors were compared (*n* = 8, *P* = .202). *P < 0 .01, NS: not significant

We investigated the expression of P-cadherin in primary and metastatic tumors. Samples were obtained simultaneously during surgical treatment. In crude analysis, there was no significant difference in P-cadherin scores of primary and metastatic lesions (data not shown). As shown in Table 2, since high expression of P-cadherin in primary lesions was significantly correlated with peritoneal dissemination, but not with lymph node metastasis, we compared P-cadherin scores of primary lesions with disseminated lesion or metastatic lymph nodes, respectively. In the comparison between primary tumors and disseminated lesions, there was no difference in P-cadherin scores (Fig. 4a). On the other hand, P-cadherin expression was reduced in metastatic lymph nodes when compared with primary tumors. (Fig. 4b).

**Table 2:** Clinicopathological characteristics of the 162 patients in association with P-cadherin expression

	P-cadherin		
Variable	Low	High	*P-*value
Age, mean ± SD (years)	55.8 ± 11.5	55.7 ± 12.9	.428
PS (no.)			.627
0	52	67	
1	11	20	
2 or 3	4	8	
CA125 ^a^(no.)			.262
≤136	37	42	
> 136	30	51	
NA	0	2	
Stage ^b^(FIGO2014) (no.)			.0024*
Stage I/II	46	42	
Stage III/IV	21	53	
Peritoneal dissemination (no.)			2.7e-5*
Positive	18	58	
Negative	49	37	
Lymph node metastasis ^b^(no.)			.823
Positive	9	15	
Negative	58	80	
Distant site metastasis (no.)			.048*
Positive	1	9	
Negative	65	86	
Histologic type^b^ (no.)			1.37e-10*
Clear cell	30	4	
Endometrioid	9	21	
Mucinous	12	11	
Serous	16	59	

Clinicopathological characteristics of the 162 patients in association with P-cadherin expression

*P < 0.05. ^a^Cutoff value as the median value. ^b^Pathological diagnosis. *NA* not available

### Recurrent lesion demonstrated equivalent P-cadherin expression to primary lesion in small group of samples

To evaluate the change of P-cadherin expression in primary and recurrent lesions, we analyzed samples of primary and recurrent lesions in eight cases. Crude analysis of eight cases demonstrated equivalent P-cadherin scores in primary tumors and recurrent lesions (Fig. 4c). Of the eight cases, two presented with either no or low P-cadherin in primary tumors and recurrent foci. Six cases of endometrioid or serous subtypes presented with similar P-cadherin scores in recurrent foci and primary tumors (Table S1). Five cases with recurrence at the peritoneum demonstrated similar P-cadherin scores with primary tumors. These results suggest that the P-cadherin expression level is predictable from the primary tumor, although this is based on a small sample size, and so necessitates further investigation.

## Discussion

This is the first study to demonstrate the expression profile of P-cadherin in ovarian cancer patients from the viewpoint of histology and tumor distribution for target therapy. First, we documented that P-cadherin was strongly corelated with unfavorable prognostic factors, with a trend toward poor survival. Second, we showed that P-cadherin is intensely and broadly expressed in specific tissue subtypes. Third, we showed that the expression pattern of P-cadherin in the primary lesion was preserved in the disseminated lesions and probably in recurrent lesions, but was reduced in metastatic lymph nodes. Together, these findings will shed light on the future development of drug-delivery strategies targeting P-cadherin in advanced ovarian cancer.

The most important finding of this study was that we clarified the expression profile of P-cadherin in ovarian cancer patients. In endometrioid and serous subtypes, 21/30 (70%) and 59/75 (78.7%) were classified as “High”, with a total immunohistochemistry score of 4 or more, whereas 18/34 (52.9%) of the clear cell subtype did not express P-cadherin at all and 30/34 (88.2%) were classified as “Low” in primary lesions. These results indicate that patients with specific subtypes of ovarian cancer are candidates for P-cadherin-targeted treatment. Furthermore, when P-cadherin expression in metastatic foci was evaluated, interestingly, corresponding disseminated foci showed similar staining to the primary foci, but it was decreased in metastatic lymph nodes (Fig. 4). Decreased E-cadherin and increased P-cadherin, called cadherin switching, has been observed when cancer progresses from FIGO stages I to II, when cancer cells spread from the primary tumor to pelvic cavity [11] {Patel, 2003 #489} [12]. Some of the mechanisms have been reported. Cheung LW et al. reported that promotions of cell migration and invasion by gonadotrophin releasing hormone (GnRH) via activation of p120 catenin signaling are mediated by the P-cadherin/insulin-like growth factor-1 receptor (IGF-1R) complex. Usui et al., using an in vitro and mouse xenograft model of ovarian cancer, demonstrated that inhibition of P-cadherin by RNAi decreased the aggregation and survival of cancer cells floating in ascites and reduced the number of peritoneal implants [3]. Taken together, these reports suggest that P-cadherin contributes to the establishment of peritoneal dissemination in ovarian cancer. Our results further indicate that P-cadherin is broadly and strongly maintained after the completion of dissemination and that P-cadherin-targeting therapy may have effects on a wide range of lesions including not only primary but also disseminated foci. From another perspective, it is suggested that biopsy of the disseminated foci acts as a substitute when biopsy of the primary lesion is difficult or highly invasive. On the contrary, P-cadherin expression was reduced in metastatic lymph nodes compared with that in concomitant primary lesions. The effect of P-cadherin-targeted therapy may not be predictable based on P-cadherin expression of the primary tumors thus, sampling is mandatory when treating patients with lymph node metastases. In fact, P-cadherin expression of dissected recurrent metastatic lymph nodes demonstrated a similar P-cadherin score to the primary tumor (Table S1), and so the patient may benefit from P-cadherin-targeted therapy. We also examined the P-cadherin expression in recurrent foci in contrast to the primary lesion. Although it is a small-scaled analysis, the P-cadherin expression pattern was maintained in the recurrent site, indicating that the results of immunohistochemistry of a primary lesion may sufficiently predict the expression of a recurrent lesion when sampling is deemed highly invasive.

Another important finding in this study was elucidation of the correlation of prognostic factors with P-cadherin in ovarian cancer patients. P-cadherin expression was significantly correlated with the histological subtype and unfavorable clinicopathologic features of ovarian cancer patients, including a high FIGO stage, positive peritoneal dissemination, and distant site metastases. Although not significant, patients with high P-cadherin expression showed a trend toward shorter DFS and OS on univariate survival analysis (Table 3, Fig. 2). When the survival analysis was limited to P-cadherin high-expressing subtypes, mucinous, endometrioid, and serous subtypes, the Kaplan-Meier survival curve demonstrated significantly decreased survival probability of DFS (*P* = 0.0264) and OS (*P* = 0.0227) in the P-cadherin High population (Fig. S1). In multivariate analysis, CA125, stage, peritoneal dissemination, and histological type, but not P-cadherin expression, were confirmed as independent prognostic factors in ovarian cancer patients. Together with clinicopathological analysis, P-cadherin may act as a confounder of other prognostic factors. As Van Marck et al. [15] reported, the impact of P-cadherin on survival differs in cancer types. In gastric [16] and oral [17] cancer, it has reported to be a good prognostic factor whereas in breast [18] [19], endometrial [20], gallbladder [21], colon [22], and pancreatic cancer [23], its overexpression indicates a poor prognosis. The fact that P-cad expression strongly correlates with unfavorable prognostic factors in ovarian cancer patients indicates that P-cadherin-targeted treatment benefits such a population, and the development of effective treatments may contribute to the improvement of survival.

**Table 3:** Univariate and multivariate analyses of predictive factors for survival of ovarian cancer patients

Factors	n	Disease-free survival				Overall survival			
		Univariate *P*-value	Multivariate			Univariate *P*-value	Multivariate		
			HR	95%CI	*P-*value		HR	95%CI	*P*-value
Age ^a^ (years)		.187				.176			
≤ 55	79								
> 55	83								
CA125^a^		6.3e-4*	2.29	1.08–4.84	.030*	.0017*	2.31	1.10–4.84	.027*
≤136	79								
> 136	81								
Stage^b^ (FIGO2014)		1.0e-7*	3.40	1.32–8.80	.011*	4.9e-7*	4.03	1.53–10.6	.0048*
Stage I/II	88								
Stage III/IV	74								
Peritoneal dissemination		5.0e-8*	4.06	1.42–11.6	.0090*	2.5e-6*	2.94	1.03–8.38	.044*
Positive	76								
Negative	86								
Lymph node metastasis		.091				.109			
Positive	24								
Negative	138								
Distant site metastasis		.062				.095			
Positive	10								
Negative	152								
Histologic type^b^		.014*	0.42	0.20–0.92	.029*	.030*	0.42	0.19–0.93	.032*
Serous	75								
Others	87								
P-cadherin		.058	1.18	0.56–2.49	.669	.050	1.19	0.55–2.58	.653
High	95								
Low	67								

Univariate and multivariate analyses of predictive factors for survival in ovarian cancer patients

*P < 0.05. ^a^Cutoff value as the median value. ^b^Pathological diagnosis. *CI* confidence interval; *HR* hazard ratio

In recent years, antibody drug therapy is being intensively researched and developed in the field of cancer treatment. Antibody therapy is largely classified in to two categories: those that act directly on functional molecules, and those that are used as drug carriers. Numerous monoclonal antibody drugs, which act on functional molecules, have been developed and have proven survival benefits. Although still in the experimental stage, the effectiveness of cell adhesion inhibition by an antibody targeting the X-dimer of P-cadherin has been reported [24]. Further advances in antibody technology have led to the rapid development of an antibody-drug conjugate (ADC) and radioimmunotherapy (RIT) that use antibodies specifically binding to cancer antigens as carriers. The first ADC approved by U.S. Food and Drug Administration (FDA) was Gemtuzumab ozogamicin for acute myelogenous leukemia (AML) in 2001 [25]. Since then, several ADC, such as Brentuximab vedotin for relapsed Hodgkin’s lymphoma (HL), systemic anaplastic large cell lymphoma (sALCL), Trastuzumab emtansine for HER2-positive metastatic breast cancer, and Inotuzumab ozogamicin for relapsed CD22-positive B-cell precursor acute lymphoblastic leukemia (ALL), have been applied in clinical practice. As for RIT, Ibritumomab tiuxetan and Iodine tositumomab, both of which use anti-CD20 to conjugate Yttrium-90 or Iodine-131, respectively, have been used to treat non-Hodgkin’s lymphoma patients [26]. Because P-cadherin shows faint expression in normal adult tissues in restricted organs such as hair follicles and the breast [27], and aberrant expression in numerous cancers [18] [19] [20] [21] [22] [23], drug-delivery systems targeting P-cadherin as a tumor-associated antigen are attractive treatment strategies. In this study, advanced ovarian cancer, especially endometrioid and serous subtypes, demonstrated intense and broad expression of P-cadherin not only in primary foci but also in disseminated lesions. Because the dissemination is the main cause of treatment failure and a poor prognosis in ovarian cancer patients, the development of ADC or RIT targeting P-cadherin may be optimal treatment to improve the prognosis of ovarian cancer patients. Although molecular targeting therapy or ADC targeting P-cadherin has yet not been developed, radionuclide bound to P-cadherin antibody has been developed. In the United States, phase 1 clinical trials have been conducted, in which Yttrium-90 bound to anti-P-cadherin antibody (FF21101) was administered to patients with solid tumors [28]. Advanced ovarian cancer, especially the endometrioid or serous subtype, may be a good candidate for such treatment.

A limitation of this study is that we could not obtain and analyze P-cadherin expression in distant metastatic lesions in the lung or liver. The small sample number of recurrent lesions is also a limitation.

## Conclusion

In this study, we revealed that P-cadherin was intensely and broadly expressed in ovarian cancer, especially in endometrioid and serous subtypes, and demonstrated preserved expression in disseminated lesions similar to that of primary foci, but not in metastatic lymph nodes. We also demonstrated that P-cadherin expression indicates a poor prognostic tendency in a confounding manner with other unfavorable prognostic factors. We believe that this study provides useful information and will help promote research and development of antibody treatment with P-cadherin as a carrier in ovarian cancer patients.

## Supplementary Information

**Additional file 1 Figure S1.** Cumulative disease-free survival and overall survival of ovarian cancer patients with mucinous, endometrioid, and serous subtypes. Significant decreases of disease-free survival (*P* = 0.0264) and overall survival (*P* = 0.0227) in the P-cadherin High expression population were observed.

**Additional file 2 Figure S2.** The original full-length blots of P-cadherin.

**Additional file 3 Figure S3.** The original full-length blots of beta-actin.

**Additional file 4 Table S1.** Pathological data and P-cadherin score of eight patients with recurrent lesions.

## Data Availability

All datasets used or analyzed for this study are available from the corresponding author upon reasonable request.

## Abbreviations

PARP: Poly ADP ribose polymerase
FIGO: International Federation of Gynecology and Obstetrics
FDA: Food and Drug Administration
IHC: Immunohistochemical staining
PBS: Phosphate-buffered salin
IS: Intensity scor
PPS: Positive percentage score
DFS: Disease-free survival
OS: Overall survival
GnRH: Gonadotropin releasing hormone
IGF-1R: Insulin-like growth factor-1 receptor
ADC: Antibody-drug conjugate
RIT: Radioimmunotherapy
AML: Acute myelogenous leukemia
HL: Hodgkin’s lymphoma
sALCL: Systemic anaplastic large cell lymphoma
ALL: Acute lymphoblastic leukemia
