# Supplementary figures and images for: Mucinous, endometrioid, and serous ovarian cancers with peritoneal dissemination are potent candidates for P-cadherin targeted therapy: a retrospective cohort study
Source: BMC Cancer. 2021 Jan 7;21:32. doi: 10.1186/s12885-020-07737-w (PMC7791827; doi:10.1186/s12885-020-07737-w)

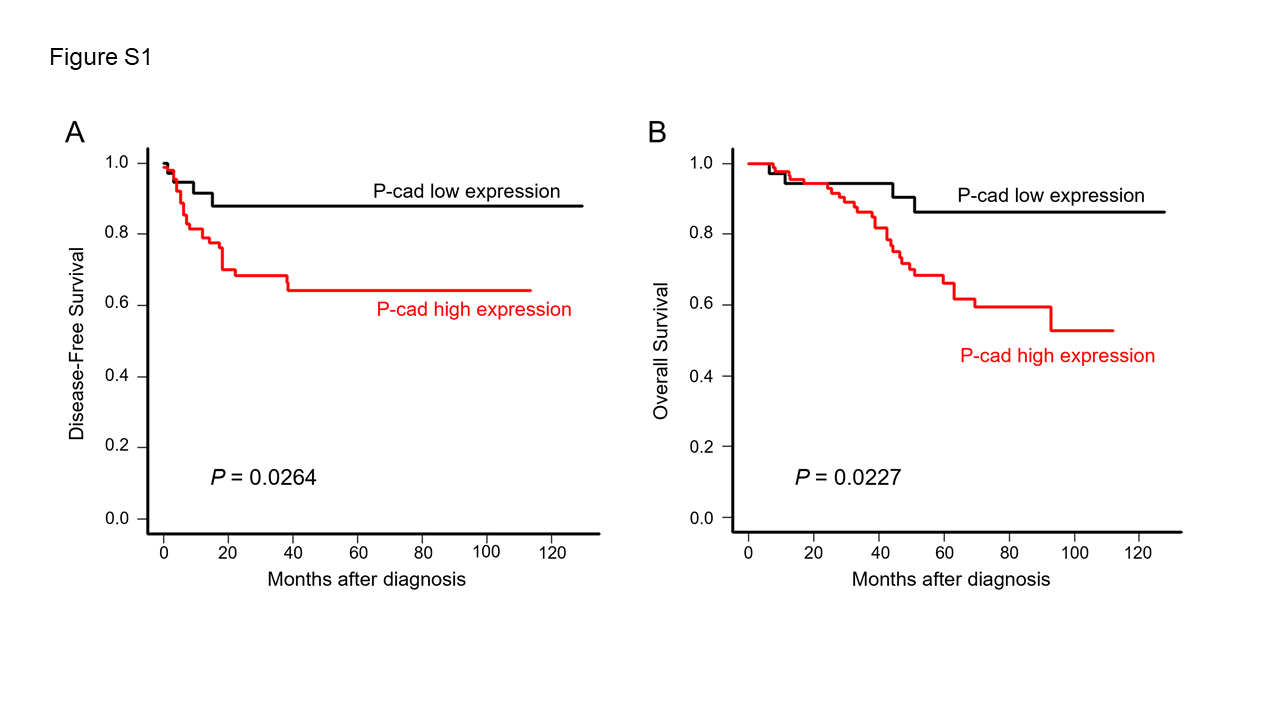

Supplement: Supplementary file 1 — Additional file 1 Figure S1. Cumulative disease-free survival and overall survival of ovarian cancer patients with mucinous, endometrioid, and serous subtypes. Significant decreases of disease-free survival (P = 0.0264) and overall survival (P = 0.0227) in the P-cadherin High expression population were observed. [file 12885_2020_7737_MOESM1_ESM.tif]

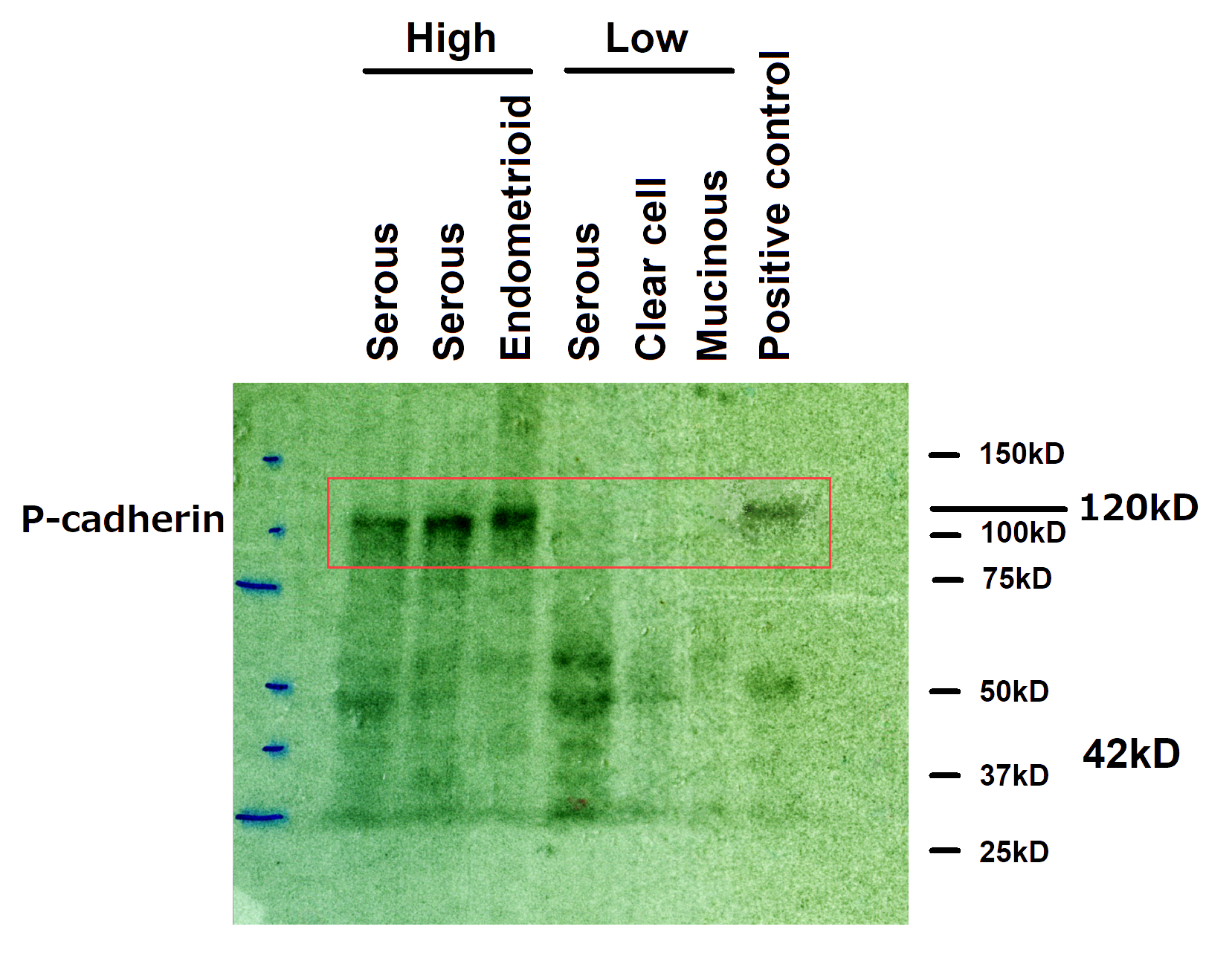

Supplement: Supplementary file 2 — Additional file 2 Figure S2. The original full-length blots of P-cadherin. [file 12885_2020_7737_MOESM2_ESM.tif]

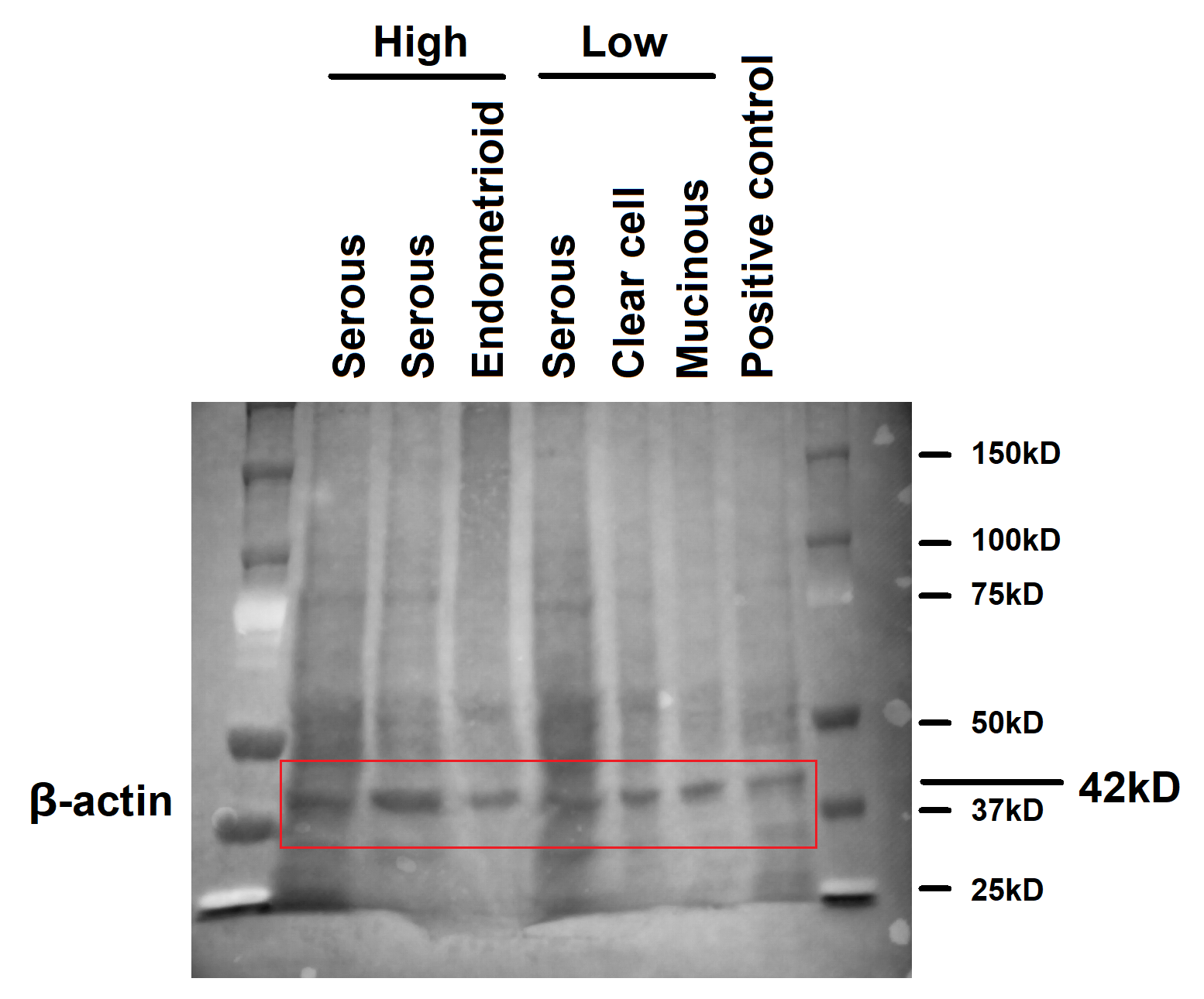

Supplement: Supplementary file 3 — Additional file 3 Figure S3. The original full-length blots of beta-actin. [file 12885_2020_7737_MOESM3_ESM.tif]
