# Supplementary material for: Mucinous, endometrioid, and serous ovarian cancers with peritoneal dissemination are potent candidates for P-cadherin targeted therapy: a retrospective cohort study
Source: BMC Cancer. 2021 Jan 7;21:32. doi: 10.1186/s12885-020-07737-w (PMC7791827; doi:10.1186/s12885-020-07737-w)
Supplement: Supplementary file 4 — Additional file 4 Table S1. Pathological data and P-cadherin score of eight patients with recurrent lesions. [file 12885_2020_7737_MOESM4_ESM.docx]

Table S1

Pathological data and P-cadherin score of eight patients with recurrent lesions

| Case | Age | Histology | Stage | Recurrent site | P-cadherin score | | |
| --- | --- | --- | --- | --- | --- | --- | --- |
|  |  |  |  |  | Primary lesion | Metastatic lesion | Recurrent lesion |
| 1 | 66 | Clear cell | ⅠC | Peritoneal dissemination | 0 | NA | 0 |
| 2 | 59 | Endometrioid | ⅡB | Peritoneal dissemination | 4 | 4 | 3 |
| 3 | 77 | Endometrioid | ⅢC | Peritoneal dissemination | 6 | 5 | 4 |
| 4 | 44 | Endometrioid | ⅢC | Inguinal lymph node | 5 | 5 | 5 |
| 5 | 48 | Mucinous | ⅠC | Peritoneal dissemination | 5 | NA | 2 |
| 6 | 68 | HGSOC | ⅡB | Peritoneal dissemination | 5 | NA | 5 |
| 7 | 73 | HGSOC | ⅢC | Ovary | 2 | 5 | 0 |
| 8 | 50 | Low-grade serous | ⅢC | Brain | 5 | 4 | 6 |

Pathological data and P-cadherin score of eight patients with recurrent lesions.

NA, not available
